# Norton’s Ophthalmic Therapeutics

**Published:** 1882-06

**Authors:** Charles Deady

**Affiliations:** Resident Surgeon New York Ophthalmic Hospital


### NORTON'S OPHTHALMIC THERAPEUTICS.

*To the Editor of* THE HOMŒOPATHIC PHYSICIAN:

In your journal for February appeared a criticism of Dr. Norton's recent work on "Ophthalmic Therapeutics," signed "G. H. C." to which I wish to take exception, as in my opinion it can hardly be called fair, and certainly does not indicate that the writer's knowledge of the subject is especially profound. Although entirely unacquainted with "G. H. C.," even by reputation, I will hazard the opinion that he does not shine as an ophthalmologist to any remarkable extent.

I wish to have it distinctly understood in starting that I am no lover of external applications, but a firm believer in the efficacy of the indicated remedy, and perfectly willing to give it in a high potency and follow it with Sac. Lac. if such shall appear to be the best treatment for the case. From this standpoint I do not feel disposed to take issue with "G. H. C." in his condemnation of local applications in general, but would only remind him that in a large dispensary practice we occasionally meet with cases with so little vitality that they fail to react to even the best selection of remedies in any and all potencies, and that sometimes in such cases an external application seems to start a reaction which we can afterwards follow up with internal medication. Our critic becomes slightly mixed when speaking of the treatment of catarrhal conjunctivitis, for he says, after quoting Dr. Norton's prescription of Zinc. sulph. and Sodium chloride, "We are even more surprised as we turn to this, for the best old-school authorities would not resort to such a lawless method," and quotes Wells as such authority, notwithstanding the fact that on page 57, in Wells' article on catarrhal ophthalmia, he distinctly states that "When the acute symptoms of irritation have subsided and those of catarrhal ophthalmia begin to show themselves astringents must be applied."

In speaking of gonorrhœal ophthalmia "G. H. C." takes exception to the abortive treatment, although this disease is not idiopathic, but the result of the local application of a poisonous virus, which should be treated in the same manner as any other poison. Here he states that "With perfect cleanliness and the indicated homœopathic remedy, one need have no fear of the result, and this we venture after treating *several cases* with success *even in the early days of our practice.*" What a tremendous *early* practice this gentleman must have had to have included in it *several* cases of conjunctivitis gonorrhœa. In the New York Eye Infirmary there were treated in eleven years 70,612 cases of eye disease, and of these only ***59*** were cases of gonorrhœal ophthalmia, or ***1*** in about ***1196***. In the New York Ophthalmic Hospital, in the eleven years from 1871 to 1881 inclusive, there were treated 32,496 eye cases, of which only ***11*** were of gonorrhœal conjunctivitis, or ***1*** to ***2954***. Is it possible that our critic is unaware of the fact that *all* inflammations of the conjunctiva occurring in the course of a gonorrhœa are *not* gonorrhœal ophthalmia?

Again, in speaking of the application of cold to the eye, he says, "Did we not know that this comes from one who is a professor in the New York Ophthalmic Hospital, we could readily believe, when we see him advising cold compresses to the inflamed eye, that he belonged to the 'regulars,' except that the 'regulars' *now* use warm, not cold applications." This is sheer nonsense. What has "pathy" to do with the application of water or ice to inflamed surfaces. In the next sentence he says, "We can here again speak from experience, and say that heat is always best to apply to any acute inflammation. The later works of the allopaths acknowledge this." To what "later" works does "G. H. C." refer? His favorite Wells is as late as anything, the last edition bearing date of 1880, and in that volume, if the doctor will read a little further than he found necessary to gather his "points," ice or cold compresses are recommended on pages 66, 73, 76, 79, 88, 137, 166, 211, 752, 791, 797 and 809.

If he is now dissatisfied with Wells, let him refer to DeWecker's "Ocular Therapeutics," pages 51, 53, 64, 66, 69, 81, 87, 165 and 433; or to Schweigger, pages 256, 263, 271, 295 and 328, where he will find the same treatment advised. In the "Handbuch der Gesammten Augenheilkunde," by Græfe and Sæmisch, the standard work on the eye in German literature, Professor Sæmisch distinctly recommends ice or cold compresses as the best treatment for conjunctival diseases. Professor Zehender emphasizes the same statement in his "Handbuch der Gesammten Augenheilkunde," bearing date of 1874, and reiterates it in 1879 in the "Lehrbuch der Augenheilkunde fur Studirende."

Finally, if the doctor will refer to Knapp's "Archives of Ophthalmology," volume 2, No. 1, dated April, 1882, he will find the following statement: "I speak now of ophthalmia neonatorum, blennorrhoic or gonorrhoic ophthalmia of the adult, of croupous and of diphtheritic ophthalmia; also of the acute stage or acute paroxysms of trachoma. The treatment of all these cases is the same, and amounts to this: *so long as the disease is on the increase or at its height, abstinence from all but indifferent local remedies, the methodical and uninterrupted application of cold, and careful cleansing.*"

This statement is from the pen of Dr. Knapp himself, the foremost old-school ophthalmologist in the United States, and perhaps the equal of any in the world. Hoping that in the spirit of fairness you will give this somewhat lengthy communication a place in your columns, I remain, Yours fraternally,

CHARLES DEADY, M. D.,
*Resident Surgeon New York Ophthalmic Hospital.*

